# Minimally invasive interventions for biopsy of malignancy-suspected pulmonary nodules: a systematic review and meta-analysis

**DOI:** 10.1590/1516-3180.2022.0543.R1.01022023

**Published:** 2023-04-17

**Authors:** André Miotto, João Aléssio Juliano Perfeito, Rafael Leite Pacheco, Carolina de Oliveira Cruz Latorraca, Rachel Riera

**Affiliations:** I IMD, PhD. Thoracic Surgeon, Assistant Professor, Thoracic Surgery Division, Universidade Federal de São Paulo (UNIFESP), São Paulo (SP), Brazil.; II MD, PhD. Thoracic Surgeon, Associate Professor, Thoracic Surgery Division, Universidade Federal de São Paulo (UNIFESP), São Paulo (SP), Brazil.; III MD, PhD. Physician, Professor, Centro Universitário São Camilo, São Paulo (SP), Brazil; Researcher, Center of Health Technology Assessment, Hospital Sírio-Libanês São Paulo (SP), Brazil; Researcher, DCenter of Health Technology Assessment, Associação Paulista para o Desenvolvimento da Medicina (SPDM), São Paulo (SP), Brazil.; Center of Health Technology Assessment, Hospital Sírio-Libanês, São Paulo, SP, Brazil; Associação Paulista para o Desenvolvimento da Medicina, Center of Health Technology Assessment, São Paulo, SP, Brazil; IV PhD. Psychologist, Researcher, Center of Health Technology Assessment, Associação Paulista para o Desenvolvimento da Medicina (SPDM), São Paulo (SP), Brazil.; 5 MD, PhD. Physician, Adjunct Professor, Discipline of Evidence-Based Medicine, Universidade Federal de São Paulo (UNIFESP), São Paulo (SP), Brazil; Coordinator, Center of Health Technology Assessment, Hospital Sírio-Libanês São Paulo (SP), Brazil.; Hospital Sírio-Libanês, Center of Health Technology Assessment, São Paulo, SP, Brazil

**Keywords:** Biopsy, Lung neoplasms, Multiple pulmonary nodules, Safety, Diagnosis, Lung cancer, Pulmonary nodules, Systematic review

## Abstract

**BACKGROUND:**

Imaging tests are important for diagnosis during the management of pulmonary nodules; however, biopsy is required to confirm the malignancy.

**OBJECTIVES:**

To compare the effects of different techniques used for the biopsy of a pulmonary nodule.

**DESIGN AND SETTING:**

Systematic review and meta-analysis were conducted using Cochrane methodology in São Paulo, São Paulo, Brazil.

**METHODS:**

We conducted a systematic review of randomized controlled trials (RCTs) on minimally invasive techniques, including tomography-guided percutaneous biopsy (PERCUT), transbronchial biopsies with fluoroscopy (FLUOR), endobronchial ultrasound (EBUSR), and electromagnetic navigation (NAVIG). The primary outcomes were diagnostic yield, major adverse events, and need for another approach.

**RESULTS:**

Seven RCTs were included (913 participants; 39.2% female, mean age: 59.28 years). Little to no increase was observed in PERCUT over FLUOR (P = 0.84), PERCUT over EBUSR (P = 0.32), and EBUSR over NAVIG (P = 0.17), whereas a slight increase was observed in NAVIG over FLUOR (P = 0.17); however, the evidence was uncertain. EBUSR may increase the diagnostic yield over FLUOR (P = 0.34). PERCUT showed little to no increase in all bronchoscopic techniques, with uncertain evidence (P = 0.02).

**CONCLUSION:**

No biopsy method is definitively superior to others. The preferred approach must consider availability, accessibility, and cost, as safety and diagnostic yield do not differ. Further RCTs planned, conducted, and reported with methodological rigor and transparency are needed, and additional studies should assess cost and the correlation between nodule size and location, as well as their association with biopsy results.

**SYSTEMATIC REVIEW REGISTRATION:**

PROSPERO database, CRD42018092367 -https://www.crd.york.ac.uk/PROSPERO/display_record.php?RecordID=92367.

## INTRODUCTION

Lung cancer is the leading cause of cancer-related deaths worldwide.^1,2^ Imaging tests are important for the diagnostic suspicion and risk evaluation of pulmonary nodules; however, biopsy is needed to confirm the malignancy.^1,2^ The technique of choice should have the highest accuracy, good diagnostic yield, and acceptable complication rate.

The minimally invasive techniques currently used include transthoracic approaches, such as percutaneous computed tomography-guided biopsy (PERCUT), and transbronchial approaches performed by bronchoscopy, such as fluoroscopy-guided transbronchial biopsies (FLUOR), transbronchial biopsies guided by endobronchial radial probes (EBUSR), and transbronchial biopsies guided by electromagnetic navigation (NAVIG).^3–7^

Mapping the literature on the comparative effects of different techniques is essential to better inform the clinicians for handling pulmonary nodules. With this evidence, better decisions can be made by incorporating the aspects of availability and affordability.

## OBJECTIVES

To identify, critically evaluate, and synthesize evidence regarding the effects of different minimally invasive techniques for the biopsy of malignancy-suspected pulmonary nodules. We aimed to highlight the benefits and harms of these techniques in comparison with each other according to the results of randomized controlled trials (RCTs).

## METHODS

We conducted a systematic review following the recommendations of the Cochrane Handbook for Systematic Reviews of Interventions^8^ and reported them in accordance with the Preferred Reporting Items for Systematic Reviews and Meta-Analyses (PRISMA) Statement.^9^ The protocol was prospectively registered in PROSPERO database (CRD42018092367, https://www.crd.york.ac.uk/PROSPERO/display_record.php?RecordID=92367) and no changes were made from the protocol since then.

### Types of studies

Only RCTs were eligible for inclusion. We included studies regardless of their status (full text or abstract), date, and language of publication.

### Types of participants

Adults with malignancy-suspected peripheral pulmonary nodules, defined as those > 8 mm and < 30 mm, with characteristics such as spiculation, pleural retraction, and growing size.^2^

### Types of interventions and comparators

- PERCUT;
- FLUOR;
- EBUSR;
- NAVIG.

RCTs comparing different sizes of bronchoscopes, nodule localization techniques, or a combination of two or more techniques were not considered.

### Outcomes of interest

#### The following primary outcomes were considered:

- Diagnostic yield was measured as the proportion of biopsies that defined the histological diagnosis of pulmonary nodules.
- Major adverse events were measured as the frequency of participants who experienced at least one major complication event, such as pneumothorax and hemothorax (symptomatic and/or requiring drainage), and death.
- The need for another technique, measured as the frequency of participants requiring further biopsy.

The following secondary outcomes were considered:

- Non-serious adverse events were measured as the frequency of at least one non-serious event, including pain.
- Time of procedure, measured in hours.
- All time points of outcome measurement were considered.

#### Search strategy

Comprehensive searches were performed in the following electronic databases or sources: CINAHL (Cumulative Index to Nursing and Allied Health Literature), Cochrane Library (via Wiley), Embase (via Elsevier), LILACS (Latin American and Caribbean Health Sciences Literature, via BVS), and MEDLINE (Medical Literature Analysis and Retrieval System Online, via PubMed). Additional searches were conducted on two clinical trial registry platforms: Clinicaltrials.gov and the WHO International Clinical Trials Registry Platform [ICTRP]) and OpenGrey (https://opengrey.eu). Manual searches were performed by screening the reference lists of included studies. All databases were searched from their inception until May 17, 2021. The search strategy is described in **Supplementary material 1** - https://drive.google.com/drive/folders/1lSHRxvUWz_Vr-cWqj3v3UFS4Nl3Z4-6K.

#### Study selection and data extraction

The study selection was performed in two phases. First, the titles and abstracts identified through the search strategy were evaluated by pre-selecting potentially eligible studies. Second, the full text was assessed to confirm the eligibility. The selection process was carried out using the Rayyan platform (https://www.rayyan.ai/)^10^ independently by two reviewers, and a third reviewer resolved any disagreements. The full selection process is detailed in the PRISMA flow diagram.

Data extraction was independently performed by two reviewers using the data extraction form, and a third reviewer resolved the disagreements.

#### Risk of bias assessment

To evaluate the risk of bias, seven domains of the Cochrane Risk of Bias (RoB) tool were used (sequence generation, allocation concealment, blinding of participants and personnel, blinding of outcome assessors, incomplete data, selective reporting, and other bias), which were classified as high, low, or unclear.^8^ Two authors independently conducted the evaluation, and a third author resolved the disagreements. The third, fourth, and fifth domains were assessed at the outcome level.

#### Data analyses

Quantitative data synthesis was performed on the results of these clinically and methodologically homogeneous studies through meta-analyses with random effect models, using the Review Manager 5.4.1 (RevMan 5.4.1) software (The Cochrane Collaboration, London, England, 2020).^8^ Relative risk (RR) and mean difference (MD) were used to estimate the effect size of dichotomous and continuous variables, respectively. A 95% confidence interval (CI) was used for all the estimates. When quantitative data synthesis was not possible, the results were reported narratively, considering whenever available, effect size estimates (including RR, absolute risk difference, odds ratio, and number needed to treat [NNT]) and their respective measures of confidence and variance (dispersion measures, CI, and P values).

Inconsistency (statistical heterogeneity) was evaluated by visual inspection of forest plots, and chi-square tests; P > 0.10 was considered indicative of statistical heterogeneity. Additionally, I² tests were used to measure the extent of inconsistency (I² > 50% was considered to indicate significant inconsistency).^9^ We explored the reasons for heterogeneity by conducting subgroup and sensitivity analyses. When necessary, the authors were contacted to obtain missing data on the outcomes of interest.

#### Additional analyses

For subgroup analyses, different anatomical regions of the nodules (central or peripheral) were explored, as different diagnostic yields were expected for each technique. Bronchoscopy methods tended to present better results in central lesions, and the transthoracic approach tended to present better yields in peripheral lesions. Sensitivity analyses were performed according to the risk of bias of the included studies (low risk versus high/unclear risk), considering the high/unclear risk of bias in at least one domain of the Cochrane RoB tool.

#### Evidence certainty

The Grading of Recommendations, Assessment, Development and Evaluations (GRADE)^12^ approach was used to assess the certainty of the body of evidence (high, moderate, low, or very low) for all comparisons. The certainty of evidence was downgraded owing to methodological limitations, inconsistencies, indirectness, imprecision, and publication bias. We developed a summary of the findings table using an online software (GRADEpro Guideline Development Tool [Software]. McMaster University, Ontario, Canada, 2022).

## RESULTS

**Figure 1:**
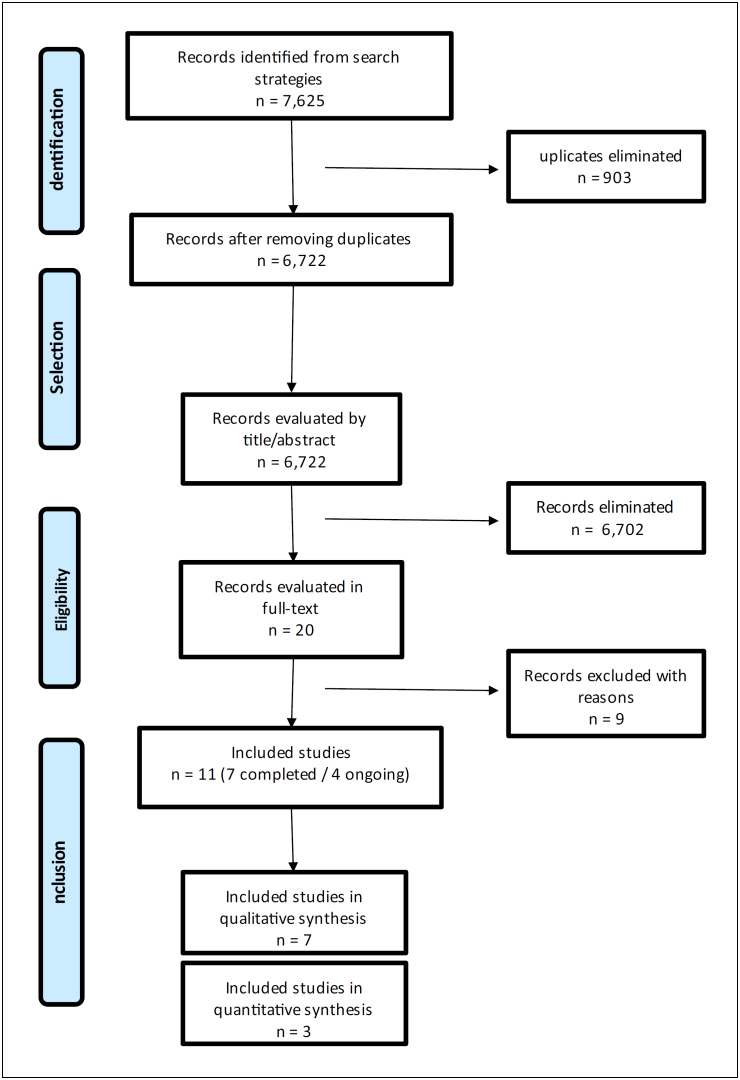
Study selection flowchart.

The search strategy retrieved 7,625 references. After removing 903 duplicates, 6,722 references were screened by title and abstract (first phase), of which 6,702 references were eliminated because they did not fulfill the eligibility criteria and 20 references were pre-selected for the second phase. After full-text reading, 11 RTCs were included: seven completed RCTs^6,^ ^11,^ ^13–17^ and four ongoing RCTs.^18–21^ The list of the nine excluded studies^22–30^ and reasons for exclusion are presented in **Supplementary material 2** - https://drive.google.com/drive/folders/1lSHRxvUWz_Vr-cWqj3v3UFS4Nl3Z4-6K. A flowchart of the study selection process is shown in Figure 1.

### Characteristics of included studies

Seven completed RCTs included in this study were published from 1998 to 2018, which included a total of 913 participants (39.2% female, n = 357) with a mean age of 59.28 years.^6,11,13–17^ All the participants had pulmonary nodules up to 3 cm on chest computed tomography without a definitive diagnosis. All RCTs reported a diagnostic yield and were considered to yield a positive biopsy when there were benign or malignant findings in the anatomopathological results. If the result was non-specific, the biopsy was considered negative and a comparison technique was performed sequentially. The main characteristics of the RCTs are shown in Table 1. Ongoing RCTs are detailed in **Supplementary material 3** - https://drive.google.com/drive/folders/1lSHRxvUWz_Vr-cWqj3v3UFS4Nl3Z4-6K.

**Table 1:** Main study characteristics

	Population	Study type	Intervention (versus control group)	Participants	Controls	Diagnostic yield	Major complications
Asano et al.^13^	Adults with suspected pulmonary nodules	RCT	NAVIG versus FLUOR	167	167	67.1% versus 59,9%	2.39% versus 1.79%
Eberhardt et al.^11^	Adults with suspected pulmonary nodules	RCT	NAVIG versus EBUSR	39	39	59% versus 69.23%	5% versus 5%
Gupta et al.^14^	Adults with suspected pulmonary nodules	RCT	EBUSR versus PERCUT	25	25	72% versus 84%	48% versus 36%
Paone et al.^15^	Adults with suspected pulmonary nodules	RCT	EBUSR versus FLUOR	87	119	75.8% versus 52.1%	0% versus 8.4%
Shankar et al.^16^	Adults with suspected pulmonary nodules	RCT	PERCUT versus FLUOR	16	18	78% versus 75%	0% versus 0%
Steinfort et al.^17^	Adults with suspected pulmonary nodules	RCT	EBUSR versus PERCUT	32	19	78.12% versus 81.25%	3% versus 20%
Wang et al.^5^	Adults with suspected pulmonary nodules	RCT	EBUSR versus PERCUT	80	80	65% versus 85%	6.25% versus 25%

RCT = randomized controlled trial; NAVIG = electromagnetic navigation transbronchial biopsy; FLUOR = fluoroscopy-guided transbronchial biopsy; EBUSR = endobronchial ultrasound with radial probe transbronchial biopsy; PERCUT = tomography-guided percutaneous biopsy.

### Risk of bias

**Figure 2:**
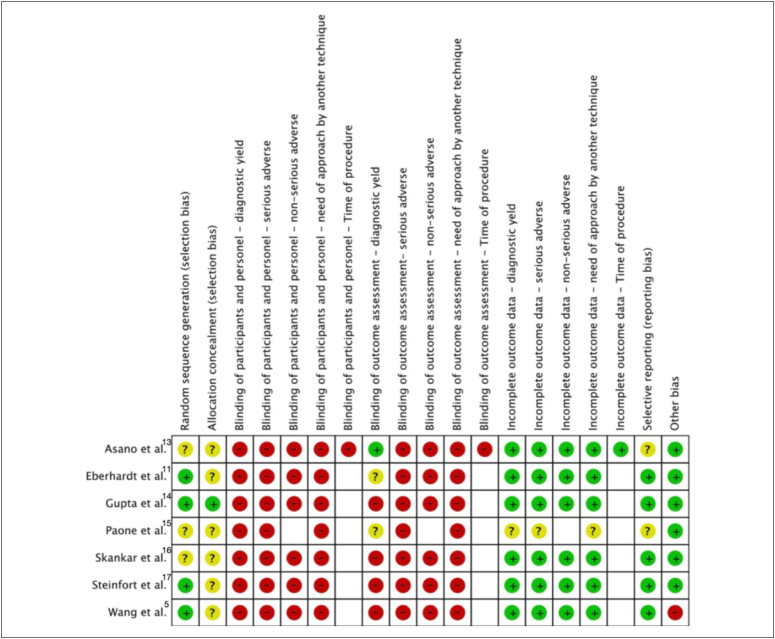
Risk of bias assessment. Summary of the risk of bias in the randomized controlled trials included for each domain.

The risk of bias of the RCTs, as assessed using the Cochrane RoB tool, is summarized in Figure 2. The reasons for each judgement are in **Supplementary material 4** - https://drive.google.com/drive/folders/1lSHRxvUWz_Vr-cWqj3v3UFS4Nl3Z4-6K. All the RCTs presented at least one domain that was judged to have a high risk of bias.

### Effects of interventions

#### Comparison 1: PERCUT versus FLUOR

One RCT assessed this comparison^11^ and the following results were found:

- Diagnostic yield: There was no difference between PERCUT and FLUOR; however, the CI was wide (RR, 1.04; 95% CI, 0.71 to 1.51), and the effect estimate was imprecise (P = 0.84; 34 participants; one RCT; very low evidence certainty) (**Supplementary material 5** - https://drive.google.com/drive/folders/1lSHRxvUWz_Vr-cWqj3v3UFS4Nl3Z4-6K).
- Need for another technique: There was no difference between FLUOR and PERCUT; however, the CI for effect estimate was wide (RR, 0.22; 95% CI, 0.03 to 1.79) and the effect estimate was imprecise (P = 0.15; 34 participants; one RCT; very low evidence certainty).

Major adverse events: No adverse events were reported in either group.

Non-serious adverse events: Two non-serious adverse events were reported in the PERCUT group and none in the FLUOR group (RR, 4.47; 95% CI, 0.23 to 86.7; 34 participants; one RCT; very low evidence certainty). In both cases, the patient had small-volume pneumothorax that was not observed during the conservative treatment, and no further intervention was necessary. There was little to no increase in safety in FLUOR compared with PERCUT; however, the effect estimate was imprecise (P = 0.32).

#### Comparison 2: PERCUT versus EBUSR

**Figure 3:**
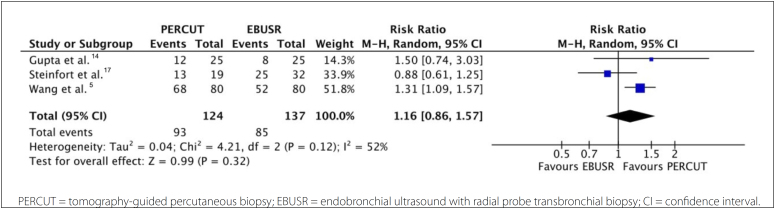
Comparison between PERCUT (CT-guided percutaneous biopsy) and EBUSR (radial probe endobronchial ultrasound-guided transbronchial biopsy) in relation to the diagnostic yield of each procedure.

Three RCTs assessed this comparison^5,15,16^ and the following results were found:

- Diagnostic yield: There was no difference between PERCUT and EBUSR; however, the CI for the effect estimate was wide (RR, 1.16; 95% CI, 0.86 to 1.57; I^2^ = 52%) and the effect estimate was imprecise (P = 0.32; 258 participants; three RCTs; very low evidence certainty) (Figure 3, **Supplementary material 6** - https://drive.google.com/drive/folders/1lSHRxvUWz_Vr-cWqj3v3UFS4Nl3Z4-6K).
- Need for another technique: There was no difference between EBUSR and PERCUT; however, the CI for effect estimate was wide (RR, 0.74; 95% CI, 0.31 to 1.77; I^2^ = 60%), and the effect estimate was imprecise (P = 0.51; 258 participants; three RCTs; very low evidence certainty).

Major adverse events: There were no differences between PERCUT and EBUSR. The CI for the effect estimate was wide (RR, 2.13; 95% CI, 0.51 to 8.99; I^2^ = 81%), and the effect estimate was imprecise (P = 0.30; 258 participants; three RCTs; very low evidence certainty).

- Non-serious adverse events: PERCUT may result in a higher risk of non-serious adverse events, with a slight increase in the estimate (P = 0.02; 258 participants; three RCTs; low evidence certainty).

#### Comparison 3: FLUOR versus EBUSR

**Figure 4:**
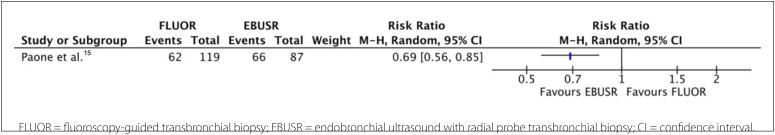
Comparison between PERCUT (CT-guided percutaneous biopsy) and bronchoscopic techniques, in relation to the diagnostic yield of each procedure.

One RCT assessed this comparison^14^ and following results were found:

Diagnostic yield: FLUOR may result in a reduction in diagnostic yield (RR, 0.69; 95% CI, 0.56 to 0.85; 206 participants; one RCT; low evidence certainty; P ≤ 0.05) (Figure 4, **Supplementary material 7** - https://drive.google.com/drive/folders/1lSHRxvUWz_Vr-cWqj3v3UFS4Nl3Z4-6K).

- Need for another technique was higher in the FLUOR group (RR, 1.98; 95% CI, 1.31 to 3.01; 206 participants; one RCT; very low evidence certainty; P ≤ 0.05).

Major adverse events: There was an increase in the risk of major adverse events with FLUOR; however, the CI for the effect estimate was wide (RR, 15.40; 95% CI, 0.91 to 259.31; 206 participants; one RCT; low evidence certainty; P ≤ 0.05).

Non-serious adverse events: No such events occurred in either group.

#### Comparison 4: FLUOR versus NAVIG

One RCT assessed this comparison^16^ and following results were found:

Diagnostic yield: There was a slight increase in NAVIG compared to FLUOR (RR, 0.89; 95% CI, 0.76 to 1.05; 334 participants; one RCT; low evidence certainty; P = 0.17) (**Supplementary material 8** - https://drive.google.com/drive/folders/1lSHRxvUWz_Vr-cWqj3v3UFS4Nl3Z4-6K).

Need for another technique: There was a slight increase in NAVIG compared to FLUOR; however, the effect estimate was imprecise (RR, 1.22; 95% CI, 0.92 to 1.62; 334 participants; one RCT; very low evidence certainty; P = 0.17).

Major adverse events: There was a slight increase in NAVIG compared to FLUOR, the CI for effect estimate was wide (RR, 0.75; 95% CI, 0.17 to 3.34), and the effect estimate was imprecise (P = 0.70; 334 participants; one RCT; low evidence certainty).

Non-serious adverse events: There were no non-serious adverse events.

- Procedure time (in minutes): No difference was observed between the interventions (MD = -3.00; 95% CI, 45.90 to 39.90; 334 participants; one RCT; low evidence certainty; P = 0.89).

#### Comparison 5: NAVIG versus EBUSR

One RCT assessed this comparison^17^ and following results were found:

- Diagnostic yield: There was no difference between EBUSR and NAVIG; however, the CI was wide (RR, 1.17; 95% CI, 0.84 to 1.64), and the effect estimate imprecise (P = 0.34; 78 participants; one RCT; very low evidence certainty) (**Supplementary material 9** - https://drive.google.com/drive/folders/1lSHRxvUWz_Vr-cWqj3v3UFS4Nl3Z4-6K).
- Need for another technique: There was no difference between EBUSR and NAVIG; however, the CI for the effect estimate was wide (RR, 0.75; 95% CI, 0.41 to 1.37), and imprecise (P = 0.34; 78 participants; one RCT; very low evidence certainty).

Major adverse events: Two patients in each group developed pneumothorax and underwent pleural drainage. No significant difference was observed (RR, 1.00; 95% CI, 0.15 to 6.75; 78 participants; RCT; very low evidence certainty; P = 1.00).

Non-serious adverse events: None were reported in any group.

Subgroup analysis (considering the location of the nodule: peripheral versus central), sensitivity analysis (considering the risk of bias: low versus high/unclear), and publication were not conducted because of the scarcity of available data assessed or reported by the included RCTs and the low number of RCTs included in a unique meta-analysis (less than ten).

### Post-hoc analysis

In clinical practice, we believe it would be interesting to have an additional comparison of PERCUT versus any other bronchoscopic technique for diagnostic yield. There was no difference between PERCUT and bronchoscopic techniques; however, the CI for the effect estimate was wide and the effect estimate was imprecise (RR, 1.14; 95% CI, 0.92 to 1.42; four RCTs; 295 participants; P = 0.02) (**Supplementary material 11** - https://drive.google.com/drive/folders/1lSHRxvUWz_Vr-cWqj3v3UFS4Nl3Z4-6K).

### Analysis of the certainty of evidence

The GRADE methodology was used to assess the certainty of evidence.^19^ Overall, the certainty of evidence was considered low or very low due to methodological limitations, indirect evidence, small sample size, and a wide CI. A summary of the certainty of evidence analysis is presented in **Supplementary material 10** - https://drive.google.com/drive/folders/1lSHRxvUWz_Vr-cWqj3v3UFS4Nl3Z4-6K.

## DISCUSSION

The choice of the method or invasive diagnosis of pulmonary nodules depends on many factors, including nodule size, localization, method availability, cost, and professional expertise. This systematic review was designed to help make this choice; however, it is difficult to compare the four different types of interventions indirectly. It is also worth noting that some of the rarely known and unavailable techniques were compared. The simplest technique evaluated was FLUOR, which requires only a common fluoroscopy device and a trained specialist. Other procedures, such as PERCUT, EBUSR, and NAVIG, require more expensive and less available materials and technology. EBUSR is a complex procedure that is performed at few centers in emerging countries. NAVIG is unavailable in Brazil and its use is far from being a current reality in many countries. Considering the rational use of resources in the health system, data from this and future related studies may help in defining which methods to carry on with realistic availability, rational use of resources, and investment in the future.

To our knowledge, no systematic review has evaluated comparisons of different lung nodule biopsy methods. There are reviews considering specific comparisons carried out under different methodologies, with different study designs and combined techniques.^6,^ ^7^ Ali et al.^6^ analyzed 25 prospective and 32 retrospective studies from a total of 7,872 lesions biopsied. The diagnostic yield for the R-EBUS group (described as EBUSR in this review) was 70.6% (95% CI, 68–73.1) and was significantly higher in malignant nodules greater than or equal to 2 cm and with a patent bronchus sign on tomography. This was a large review, with many studies included; however, the certainty of evidence was lost with the inclusion of retrospective studies, which comprised the majority of included studies. Furthermore, the conclusion that patients undergoing PERCUT have higher complication rates (up to 23%) versus 2.8% for EBUSR, should be considered as having low certainty as most of the studies used this analysis were retrospective and not masked. However, Gupta et al.,^14^ a study included in our review, showed 20% pneumothorax in PERCUT, confirming the rate suggested by Ali et al.^6^ This high rate may be the result of the small number of participants in the study. Gupta et al.^14^ also showed that the diagnostic yield for nodules located in the right superior lobe was significantly lower in EBUSR.

McGuire et al.^7^ analyzed 41 prospective and retrospective studies of 2,988 involved nodules (2,102 biopsied by EBUSR and 886 biopsied by NAVIG). The methods had a complication rate of less than 2% and were considered good options for diagnosing peripheral nodules. However, the review was conducted considering a large proportion of retrospective studies, which reduced the certainty of the evidence and increased the risk of bias. Additionally, other biopsy methods were not considered.

Our search was more comprehensive and sensitive (beyond the MeSH term, we used text words and a list of synonyms for each term) with no restrictions on date, language, or status of the publication. We assessed the certainty of the evidence using the GRADE approach, which was not used in the aforementioned reviews.

The limitations of our study were primarily related to the poor methodological quality of the included RCTs. In general, the included RCTs had a high risk of bias, small sample sizes, and clinical heterogeneity. As we considered any technique, different comparisons were assessed by the included RCTs using the same technique, which made it difficult to define the best method. Further RCTs, planned, conducted, and reported with methodological rigor and transparency are needed on this issue, and additional studies should provide information about the nodule size and location and their relation to the biopsy results.

Although the techniques described in this study have been used in clinical practice, we did not find sufficient evidence to determine the preferred technique. Until more robust evidence can better support therapeutic decisions, the available evidence suggests the following:

- In the choice between PERCUT and FLUOR, there seems to be no difference between the methods regarding diagnostic yield (P = 0.84); however, PERCUT required fewer approaches using another technique (P = 0.15), and FLUOR was safer (P = 0.32). However, this benefit might not be clinically relevant.

PERCUT appears to be more advantageous than EBUSR in terms of diagnostic yield (P = 0.32) and safety for serious (P = 0.30) and non-serious (P = 0.02) adverse events. EBUSR may have a lower need for another technique (P = 0.51). However, whether this difference is clinically relevant remains unclear.

- Between FLUOR and EBUSR, EBUSR has an advantage regarding diagnostic yield, safety for serious adverse events, and a lower need for another technique (P ≤ 0.05).

No differences were observed between PERCUT and NAVIG regarding safety (P = 0.70); however, there was an advantage for NAVIG regarding diagnostic yield (P = 0.17) and a lower needfor an approach using another technique (P = 0.17). There was no difference in the procedure time between the FLUOR and NAVIG groups (P = 0.89).

- Between NAVIG and EBUSR, EBUSR appears to be advantageous regarding diagnostic yield (P = 0.34) and a lower need for the use of another technique (P = 0.34), but there was no difference regarding safety (P = 1.00). However, whether this difference is clinically relevant remains unclear.

The most recommended technique is unclear, but PERCUT and NAVIG stand out as favored techniques in most RCTs. In direct comparison, PERCUT has an advantage, although not significant because of the wide CI (P = 0.02).

A cost analysis was not performed, as only clinical trials were included. Study designs that evaluate cost-utility, effectiveness, and benefit would better assess these data. A study that evaluated the cost-effectiveness of PERCUT versus NAVIG in the United Kingdom in 2020 showed that NAVIG may be more cost-effective than PERCUT in some subgroups; however, there is no general definition of one method in relation to another, particularly if the cost of implementing NAVIG is considered.^31^

The procedures had similar risks of complications and no significant difference; however, there was still a difference. It is up to the physician to discuss each method and present the possible risks and benefits of a shared decision on the method, respecting the ethics and opinions of patients and families.

## CONCLUSION

This systematic review did not identify high-certainty evidence to support the choice of one method of lung nodule biopsy over others. In this scenario of uncertainty, until the results of new studies are published, the preferred choice of biopsy method must consider availability and accessibility. Potential risks and benefits must be presented to patients for a shared decision.
